# Interplay of mRNA capping and transcription machineries

**DOI:** 10.1042/BSR20192825

**Published:** 2020-01-24

**Authors:** Zaur M. Kachaev, Lyubov A. Lebedeva, Eugene N. Kozlov, Yulii V. Shidlovskii

**Affiliations:** 1 Laboratory of Gene Expression Regulation in Development, Institute of Gene Biology, Russian Academy of Sciences, Moscow, Russia; 2 Department of Biology and General Genetics, I.M. Sechenov First Moscow State Medical University, Moscow, Russia

**Keywords:** capping checkpoint, mRNA capping, promoter, transcription pausing, transcription

## Abstract

Early stages of transcription from eukaryotic promoters include two principal events: the capping of newly synthesized mRNA and the transition of RNA polymerase II from the preinitiation complex to the productive elongation state. The capping checkpoint model implies that these events are tightly coupled, which is necessary for ensuring the proper capping of newly synthesized mRNA. Recent findings also show that the capping machinery has a wider effect on transcription and the entire gene expression process. The molecular basis of these phenomena is discussed.

## Introduction

A characteristic feature of eukaryotic mRNAs is the presence of the cap structure at the 5′-end. This structure consists of an inverted 7-methylguanosine linked to the first-transcribed nucleotide of a newly synthesized transcript and is subsequently bound by the cap-binding protein complex (CBC). The major cap function is to stabilize nascent transcripts by protecting mRNA from 5′-exonucleases [1,2]. In addition, the cap helps to recruit factors necessary for splicing, 3′-end processing, export, and translation [3,4].

The mRNA capping machinery has three basic activities: RNA 5′-triphosphatase (RT), guanylyltransferase (GT), and RNA guanine-N7 methyltransferase (RNMT). In yeast, there are three separate enzymes responsible for these activities. The RT and GT enzymes are combined in one functional complex in *Saccharomyces cerevisiae* but function separately in *Schizosaccharomyces pombe* [5,6]. In metazoans, the first two activities are performed by the same capping enzyme (CE), while the methyltransferase activity resides in the RNMT–RAM complex in which RNMT is the catalytic subunit and RAM is the activating subunit stimulating RNMT activity [7–9]. Moreover, the capping machinery includes cap-specific mRNA (nucleoside-2′-O-)-methyltransferases CMTR1 and CMTR2 (CMTr) and also recently described cap-specific adenosine N6-methyltransferase (CAPAM) [10].

The eukaryotic mRNA capping process does not always proceed to completion [11]. In mammals, proteins of the DXO/Dom3Z and XRN families serve as surveillance proteins in 5′-end capping quality control. In particular, decapping exoribonuclease DXO degrades capped but unmethylated pre-mRNA [12,13]. In *S. cerevisiae*, there are partially redundant machineries for RNA cap quality control: Rai1-Rat1 and Ydr370C/Dxo1 [11,14–16].

Capping occurs co-transcriptionally with the capping and transcriptional machineries being tightly coupled. Below, we describe current views on the connection between the capping process and transcriptional events. Table 1 shows the list of factors participating in the establishment of this connection in different organisms.

**Table 1:** Factors participating in capping–transcription coupling

Protein/protein complex	*S. cerevisiae*, budding yeast	*S. pombe*, fission yeast	*Drosophila melanogaster*, fly	*Mus musculus*, mouse	*Homo sapiens*, human
RNA GT	Ceg1	Pce1	mRNA-cap	RNGTT	RNGTT
5′ phosphatase, RT (RNGTT)	Cet1	Pct1			
MT- RNMT	Abd1	Pcm1	l(2)35Bd	RNMT	RNMT
Cap-specific mRNA (nucleoside-2′-O-)-methyltransferase (CMTr)	-	-	CG6379, aft	CMTR1, CMTR2	CMTR1, CMTR2
CAPAM	-	-	CG11399	PCIF1	PCIF1
CBC	Sto1+cbc2	cbc1+cbc2	Cbp80+ Cbp20	NCBP1+NCBP2	NCBP1+ NCBP2
TFIIH kinase subunit	Kin28	Mcs6	Cdk7	Cdk7	Cdk7
Phosphatase of Pol II CTD (CPF)	Ssu72	Ssu72	Ssu72	Ssu72	Ssu72
Positive transcription elongation factor (P-TEFb)	Ctk1+Ctk2+ Ctk3, Bur1+Bur2	Cdk9+Pch1	Cdk9+ CycT	Cdk9+CCNT1	Cdk9+ CCNT1
DRB sensitivity-inducing factor (DSIF)	Spt4+Spt5	Spt4+Spt5	SPT4+SPT5	(SUPT4a/SUPT4b)+SUPT5	SUPT4H1+SUPT5H
Negative elongation factor (NELF)	-	-	NELFA+ NELFB+ TH1+ NELFE	NELFA+ NELFB+ NELFC/D+ NELFE	NELFA+ NELFB+ NELFC/D+ NELFE
Polyadenylate-binding protein-interacting protein 2 (Paip2)	-	-	Paip2	Paip2a, Paip2b	Paip2a, Paip2b

Abbreviations: Cdk7, cyclin-dependent kinase 7; PCIF1, cap-specific adenosine N6-methyltransferase; Pol II, RNA Polymerase II.

## Capping checkpoint model

RNA Polymerase II (Pol II)-dependent transcription is a complex process regulated by multiple factors and signals, which involves several checkpoints to ensure proper mRNA synthesis and assembly of mRNA–protein complex [17,18]. The principal checkpoints include proper mRNA capping, splicing, and 3′-end formation.

Eukaryotic transcription occurs in the following basic stages: the assembly of preinitiation complex, initiation, elongation, and termination. The initiation–elongation transition is further divided into ‘early elongation’ and ‘productive elongation.’ These two substages are separated by Pol II pausing: after transcription of approximately 20–60 nucleotides, Pol II stops the synthesis until a specific signal comes [19]. Early studies on this phenomenon on the *hsp* model genes have indicated that the pausing is connected with mRNA cap formation [20].

The capping checkpoint model suggests that Pol II pauses at promoter-proximal region to ensure mRNA capping prior to the onset of productive elongation. Arrest of early elongation ensures the recruitment of the CEs, which in turn attract other factors to cancel the arrest. Capping occurs progressively as Pol II moves through the pause region: the most proximal paused RNAs are largely uncapped, while more distal are completely capped [20]. Capping in this context means the addition of capping guanosine, while the time point of its methylation during transcription has not yet been determined with certainty. That is why the term ‘capping’ refers below to the event of guanosine addition, with cases of its methylation being specifically indicated.

**Figure 1:**
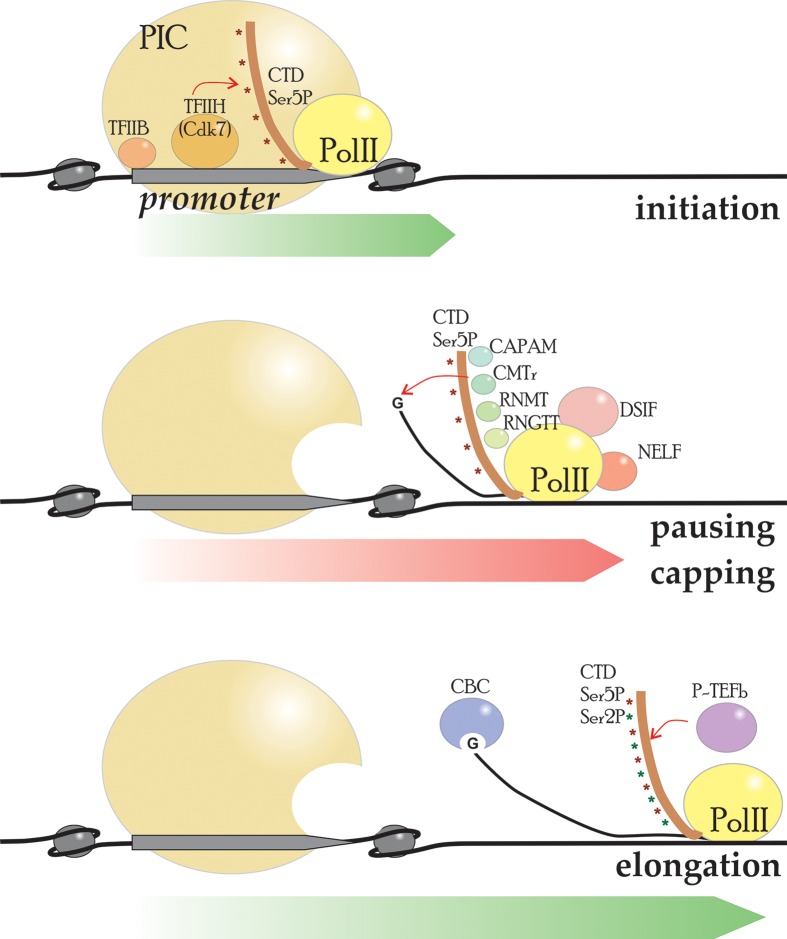
Early stages of transcription Capping and transcription factors acting jointly at early stages of transcription: initiation, pausing, and elongation. The factors crucial for transcription–capping interplay are shown. Abbreviation: PIC, preinitiation complex.

There are several factors ensuring proper transition though the pausing stage [21]. Following the transcription initiation, the DRB sensitivity-inducing factor (DSIF) binds Pol II [22,23] and recruits the negative elongation factor (NELF) on to chromatin [24]. The latter causes a transcriptional pause, during which the capping machinery is recruited. During early elongation, the Pol II C-terminal domain (Pol II CTD) is phosphorylated at the Ser^5^ residue by the cyclin-dependent kinase 7 (Cdk7), a subunit of TFIIH. Ser^5^-phosphorylated Pol II CTD and Spt5 subunit of DSIF recruit the capping machinery [25,26]. The recruitment of CE can relieve the action of NELF and provide a platform for the positive transcription elongation factor (P-TEFb) loading [27,28]. The latter phosphorylates Ser^2^ of Pol II CTD [29], DSIF [30,31], and NELF factors [32]. As a result, phosphorylated NELF dissociates and the paused Pol II is released into productive elongation (Figure 1).

## Capping machinery functioning depends on transcription

The mRNA capping apparatus and early elongation factors show tight connection in the nucleus. The central players connecting these machineries are Pol II CTD and Spt5 [28,33,34]. Indeed, Pol II with a truncated CTD displays capping defects [35]. Pol II CTD has a specific phosphorylation pattern, which depends on the stage of the transcription process [36], and the recruitment and functioning of CEs depend on this pattern [37].

It is the phosphorylated CTD that couples transcription with capping [37]. More precisely, its phosphorylation at Ser^5^ (not at Ser^2^) stimulates capping activity [38]. Ser^5^ phosphorylation on the promoter provides for correct early mRNA processing. Ser^5^-phosphorylated Pol II has been shown to co-purify with the capping machinery and TFIIH kinase [39]. Recent research has shown that Ser7 phosphorylation is also important for capping: it is necessary for GT Ceg1 association with Pol II in yeast [40].

Ser^5^-phosphorylated Pol II CTD and Spt5 in fission yeast interact with GT Pce1 and triphosphatase Pct1 [41,42]. In budding yeast, RNA GT Ceg1 binds to Ser^5^-phosphorylated CTD and recruits the Cet1 triphosphatase to Pol II, and it has also been shown that Ceg1 and Abd1 bind directly and independently to phospho-CTD [43,44].

Ser^5^ phosphorylation is also important for RNGTT binding and its activity in mammals [44,45]. Human RNMT recruitment also depends on Ser^5^ phosphorylation [46], with RNMT forming ternary complexes with the CE and the elongating form of Pol II [47]. The GT domain of mouse CE Mce1 binds to the phosphorylated CTD [44]. Moreover, the mammalian GT is activated allosterically by binding to Ser^5^-phosphorylated CTD [38].

Thus, the connection of capping machinery with Ser^5^-phosphorylated Pol II CTD is a conserved feature [38]. However, the location and composition of the CTD-binding site in the mammalian CE is distinct from that in the yeast CE, which recognizes the same CTD primary structure [45].

In budding yeast, the recruitment of all three CEs to the 5′-ends of transcribed genes requires Kin28 [25,48,49]. This is a major kinase responsible for Ser^5^ phosphorylation (an ortholog of Cdk7 subunit of TFIIH in Metazoa). Inhibition of human Cdk7 activity results in reduced CE recruitment, with consequent decrease in mRNA capping, and increased Pol II promoter-proximal pausing [50–52].

As expected, Pol II CTD phosphatases, which act during productive elongation, antagonize CE binding. Thus, phosphatase FCP1 is necessary for the dissociation of the CE from Pol II CTD in mammals [49].

However, phosphorylated Pol II CTD alone is not sufficient for efficient capping [53]. There is a CTD-independent, but Pol II-mediated mechanism that functions in parallel with CTD-dependent processes to ensure optimal capping [54]. The mRNA CE in yeast requires two interfaces for the interaction with Pol II: Ser^5^-phosphorylated CTD and the multihelical foot domain of Rpb1. The latter contributes to the specificity of CE interaction with Pol II [55]. Mutations in the foot domain or the factors associated with it lead to increase in Ser^5^ phosphorylation [56,57].

In *S. cerevisiae*, Cet1 and Ceg1 interact with Pol II in heterotetrameric Cet1_2_Ceg1_2_ form [4,58]. The interaction between Ceg1 and the phosphorylated Pol II CTD is well studied [45,59], but Cet1 also forms extensive interactions with the transcribing Pol II complex outside of CTD. Ceg1 appears to be mobile and adopt multiple conformations in the transcribing complex. Contacts with Pol II subunit Rpb7 have also been observed [58]. Thus, the combination of CTD-independent and CTD-dependent tethering mechanisms plays a dominant role in activation of co-transcriptional capping.

Another transcription factor contributing to capping machinery recruitment is the Spt5 protein, a subunit of early Pol II elongation factor DSIF. Spt5 carries a CTD that plays a role similar to that of Pol II CTD. Both Spt5 and Pol II CTDs are important for the recruitment of CEs [34]. In fission yeast, triphosphatase Pct1 and GT Pce1 bind independently to the elongation factor Spt5 [42]. In budding yeast, Spt5 contributes to stable recruitment of the mRNA CEs Cet1, Ceg1, and Abd1 [33].

There is experimental evidence for the concept of an ‘Spt5 CTD code,’ similar to that of Pol II CTD. According to this concept, the Spt5 CTD is structurally flexible and can adopt different conformations that are templated by particular cellular Spt5 CTD receptor proteins; moreover, threonine phosphorylation of the Spt5 CTD repeat inscribes a binary on-off switch that is read by diverse CTD receptors, each in its own distinctive manner [60]. Unlike with the Pol II CTD, phosphorylation of Spt5 CTD by P-TEFb blocks the Spt5–Pce1 interaction [59].

It is noteworthy that the way of CEs recruitment may be species-specific. In budding yeast, the cap methyltransferase Abd1 interacts directly with phosphorylated serine 5 CTD Pol II, whereas the cap methyltransferase Pcm1 in fission yeast is recruited in a complex with Cdk9/Pch1 (P-TEFb) [49,61], with Cdk9/Pch1 apparently targeting the capping apparatus to the transcriptional complex [61].

One more general transcription factor participates in proper capping. This is the TFIIB tip region, which is required for appropriate levels of serine 5 CTD Pol II phosphorylation and mRNA capping [62].

In mammals, the first- and second-transcribed nucleotides can also be O-2 methylated by CMTR1 and CMTR2 enzymes [63,64]. This modification has a role in translation initiation and identification of transcripts as ‘self’ in innate immunity [4]. CMTR1 is recruited to Ser^5^-phosphorylated Pol II CTD early in transcription [65].

Recently, a novel methyltransferase CAPAM was discovered. It catalyzes N6-methylation of the first-transcribed adenosine [10,66,67]. CAPAM was identified as Ser^5^-phosphorylated CTD-interacting factor 1 (PCIF1) [10,68]. While initially CAPAM was found to have a negative effect on RNA Pol II-dependent transcription of a model gene [68], later transcriptome-wide analyses showed its gene-specific effect on transcription [66,69–71].

Thus, there are a number of interactions between CEs and Pol II and Spt5. These interactions are crucial for the recruitment and functioning of CEs. The connection between the capping machinery and transcriptional apparatus is bimodal. On the one hand, Pol II CTD may serve as a scaffold that brings together the 5′-end of mRNA and CEs, thereby increasing capping efficiency; on the other hand, transcription factors may allosterically enhance the activity of the capping machinery [54].

## Capping apparatus has an effect on transcription

The relationship between CEs and transcriptional machinery is mutual [72]. Capping is enhanced by the interaction of CEs with CTD Pol II, but CEs influence transcription as well [28,73–75].

As shown initially, Cet1 inhibits transcriptional reinitiation [73] and lowers the accumulation of Pol II at the promoter-proximal region independent of mRNA capping activity [76]. Ceg1 stimulates early elongation [74,77], with its mutants being defective in this process. The latest findings show that the Cet1–Ceg1 complex and DSIF stimulate Pol II promoter escape and transition to elongation [78].

The yeast methyltransferase Abd1 has a gene-specific stimulatory effect on Pol II recruitment on to a promoter. Stimulation of transcription by Abd1 occurs in a methylation-defective mutant and is therefore independent of capping itself. Conditional mutants in Abd1 have defects in Pol II binding to the promoter at some genes and in promoter clearance or early elongation at other genes. Abd1 depletion results in hyperphosphorylation of CTD Ser^5^ [74].

The fission yeast cap-methyltransferase Pcm1 recruits P-TEFb to chromatin. When this connection is disrupted, Cdk9 is not properly recruited and Pol II elongation is severely affected [32]. Interaction of Cdk9 with RNA triphosphatase Pct1 has also been described in *S. pombe* [79].

The distribution of CEs along a gene implies that they influence later stages of transcription. In yeast, genome-wide occupancy for Cet1 and Ceg1 is restricted to the transcription start site, whereas occupancy for Abd1 peaks at 110 bp downstream, and occupancy for the CBC rises subsequently [33]. Presumably, the Ceg1–Cet1 capping apparatus dissociates from the transcription complex as Ser^5^ phosphorylation decreases during elongation, whereas the yeast Abd1 cap methyltransferase has prolonged interactions with the Pol II CTD.

Human CEs are found at 5′ ends and throughout genes, including even 3′-flanking regions more than a kilobase downstream of the poly(A) site [80]. Capping factors can therefore influence transcription elongation, termination, and 3′-end mRNA processing.

The *S. cerevisiae* Cet1 N-terminal domain (NTD) promotes the recruitment or facilitates chromatin transcription (FACT), which enhances the engagement of Pol II into transcriptional elongation on an active gene, independently of mRNA capping activity. The absence of the Cet1 NTD impairs FACT targeting and consequently reduces the engagement of Pol II in transcriptional elongation, leading to a promoter-proximal accumulation of Pol II [81].

In mammals, the CE counteracts transcriptional repression with the help of NELF [28,82]. Mammalian RNMT–RAM promotes Pol II-dependent transcription as well. The impact of RNMT–RAM on transcription is direct and independent of mRNA capping, stability, and translation. RNMT–RAM binds the full length of pre-mRNA and recruits proteins associated with transcription [75].

Thus, CEs have been found to regulate transcription independently of their enzymatic activity. The mechanism of this effect seems to be gene- and species-specific.

Another important participant of capping–transcription interplay is the CBC. It has a reciprocal relationship with early transcription events as well. CBC participates in recruiting transcription factors and regulating the transcription process [3]. CBC interacts with CTD kinases in yeast, its depletion affects phosphorylation level of CTD Ser^2^ and Ser^5^ [83]. In mammals, CBC stimulates transcription elongation via the recruitment of NELF [84] and P-TEFb and is important for CTD Ser^2^ phosphorylation [27].

In *S. cerevisiae*, CBC recruits Ctk2 or Bur2, an ortholog of P-TEFb [83]. Abd1 and CBC are important for the recruitment of kinases Ctk1 and Bur1, which promote elongation and CE release [33].

The mRNA CBC also stimulates the formation of preinitiation complex at the promoter via its interaction with Mot1p *in vivo* in *S. cerevisiae* [85]. Mot1p is a regulator of transcription that positively or negatively regulates expression of Pol II-transcribed genes in a gene-specific manner. CBC represses the weak terminator by impeding recruitment of the termination factors Pcf11p and Rna15p (subunits of the cleavage factor CFIA) [86].

Thus, CBC has multiple functions in the transcription process. Recently, polyadenylate-binding protein-interacting protein 2 has been described as a novel partner of CBC. These proteins act jointly at the early elongation stage of transcription, and their depletion affects Ser^5^ CTD Pol II phosphorylation in *Drosophila* [87,88].

## Capping as a regulatory step in gene expression

Over recent years, examples have emerged to illustrate the regulation of mRNA cap formation by different signals. These signals regulate the rate and extent of mRNA cap formation, resulting in changes in gene expression [89]. The first described case of regulated capping was c-myc-dependent activation [90]. c-Myc regulates the formation of the cap on many transcripts. c-Myc increases the recruitment of catalytically active CE to Pol II and to its target genes [91]. In addition, c-myc causes increased cap methylation, which makes a significant contribution to c-myc-dependent gene regulation, and increased cap methylation is linked to c-myc-dependent enhancement of TFIIH and P-TEFb activity [92]. Since the 7-methylguanosine cap is required for effective translation, the enhanced methyl cap formation also increases protein production from c-myc-responsive genes to a level that exceeds the transcriptional induction. Thus, the regulation of capping is a way to enhance protein synthesis regardless of the transcription augment *per se* [92,93].

Another example is the E2F1 transcription factor, which promotes the formation of the cap via a mechanism dependent on Pol II phosphorylation [93,94].

Capping is regulated by signaling pathways. For example, RNMT is phosphorylated and activated by CDK1-cyclin B1 during the cell cycle. This results in elevated cap methyltransferase activity, with transcription being reinitiated at the beginning of the postmitotic G_1_ phase. It is the way to coordinate mRNA capping with the burst of transcription [95]. In yeast, nutrient starvation causes a general decrease in cap methylation [11]. It has been reported that importin-α stimulates general cap methylation [96]. Conversely, CE mRNA-cap positively regulates Hh signaling activity through modulating PKA activity in *Drosophila* [97].

Thus, the regulatory factors of mRNA capping, together with the capping quality control mechanism, may provide yet another layer of the regulation of gene expression.

## Conclusions and prospects

The capping checkpoint model described above has been confirmed in many studies. However, the current model of capping/transcription interplay is not yet complete. There are some issues that need further investigation. An important future challenge is to clarify the cause-and-effect relationships that link capping with transcriptional pausing and elongation. The extensive network of interactions between proteins of capping and transcription machineries is not known in detail. Thus, the mechanism of CBC recruitment on to nascent mRNA is poorly understood, and the same is true of the recruitment of surveillance decapping machinery [98].

Species- and gene-specific interactions are of special interest. Capping and transcription machineries possess some species-specific features [99], e.g. there is a considerable number of metazoan-specific factors and intraspecific paralogs (Table 1). Moreover, non-catalytic regulatory domains of CEs in higher eukaryotes have high diversity even in closely related species. These facts may imply the involvement of these factors and domains in specific interactions with the transcriptional apparatus. Some examples of species-specific connections are mentioned above, including distinct sites of mammalian/yeast CE interaction with phospho-CTD Pol II, different ways of GT–RT interaction with Pol II in budding/fission yeast, the existence of NELF–CE interaction in mammals, and different targets for Abd1/Pcm1 in the transcriptional machinery.

Gene specificity in the functioning of the capping machinery has been described in several cases, such as the aforementioned c-myc- and E2F1-dependent regulation of capping. It may well be that other gene-specific transcription factors can also act in a similar way and regulate local capping [100]. Capping factors may also be a target for gene-specific signaling pathways, as is the NTD of RNMT, which carries multiple modification sites [46,95].

The capping step does not always proceed to completion. An important as yet unanswered question is whether this is simply a consequence of the intrinsic inefficiency of the capping process or a regulated event to modulate subsequent pre-mRNA processing. If the latter is true, what are the components involved, and how are the decisions made as to which pre-mRNAs are to be capped [13]?

The previous model of capping implies that capping plays a ‘passive’ role in regulating gene expression by preventing degradation of mRNAs [101]. Now it is clear that capping can be modulated to ‘actively’ regulate the fate of a bulk or a selected subset of mRNAs. It is still unclear what set of genes and transcription factors participate in regulated capping. More studies are needed to gain a deeper insight into the influence of signaling pathways on capping and the role of regulated capping in the whole context of gene expression.

## Abbreviations

CAPAM: cap-specific adenosine N6-methyltransferase
CBC: cap-binding protein complex
Cdf7: cyclin-dependent kinase 7
CE: capping enzyme
DSIF: DRB sensitivity-inducing factor
FACT: facilitates chromatin transcription
GT: guanylyltransferase
NELF: negative elongation factor
NTD: N-terminal domain
Pol II: RNA Polymerase II
Pol II CTD: Pol II C-terminal domain
P-TEFb: positive transcription elongation factor
RNMT: RNA guanine-N7 methyltransferase
RT: RNA 5′-triphosphatase
